# Sliding Rocks on Racetrack Playa, Death Valley National Park: First Observation of Rocks in Motion

**DOI:** 10.1371/journal.pone.0105948

**Published:** 2014-08-27

**Authors:** Richard D. Norris, James M. Norris, Ralph D. Lorenz, Jib Ray, Brian Jackson

**Affiliations:** 1 Scripps Institution of Oceanography, La Jolla, California, United States of America; 2 Interwoof, Santa Barbara, California, United States of America; 3 Applied Physics Laboratory, The Johns Hopkins University, Laurel, Maryland, United States of America; 4 Department of Physics, Boise State University, Boise, Idaho, United States of America; Centro de Investigacion Cientifica y Educacion Superior de Ensenada, Mexico

## Abstract

The engraved trails of rocks on the nearly flat, dry mud surface of Racetrack Playa, Death Valley National Park, have excited speculation about the movement mechanism since the 1940s. Rock movement has been variously attributed to high winds, liquid water, ice, or ice flotation, but has not been previously observed in action. We recorded the first direct scientific observation of rock movements using GPS-instrumented rocks and photography, in conjunction with a weather station and time-lapse cameras. The largest observed rock movement involved >60 rocks on December 20, 2013 and some instrumented rocks moved up to 224 m between December 2013 and January 2014 in multiple move events. In contrast with previous hypotheses of powerful winds or thick ice floating rocks off the playa surface, the process of rock movement that we have observed occurs when the thin, 3 to 6 mm, “windowpane” ice sheet covering the playa pool begins to melt in late morning sun and breaks up under light winds of ∼4–5 m/s. Floating ice panels 10 s of meters in size push multiple rocks at low speeds of 2–5 m/min. along trajectories determined by the direction and velocity of the wind as well as that of the water flowing under the ice.

## Introduction

**Figure 1:**
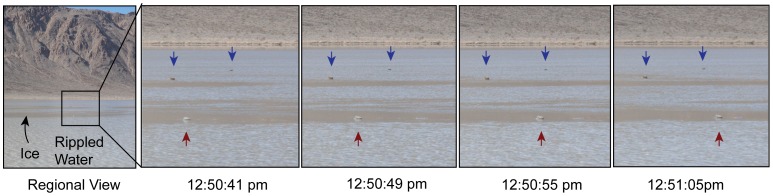
Time lapse images of a moving rock. Image acquired with a handheld digital camera on January 9, 2014. Image on the left shows the wide-angle view; interior black frame indicates the view in other frames. In close-up frames, blue arrows show stationary rocks and red arrow—a rock in motion (moving from left to right). Total movement lasted ∼18 seconds. Dark, flat areas on the pond are panels of ∼3 mm thick ice surrounded by rippled water several centimeters deep. Ice thickness estimated from inshore ice panels. Broken ice panels accumulated on the upstream side of the moving rock in the last two images. Images have been cropped but not otherwise edited.

**Figure 2:**
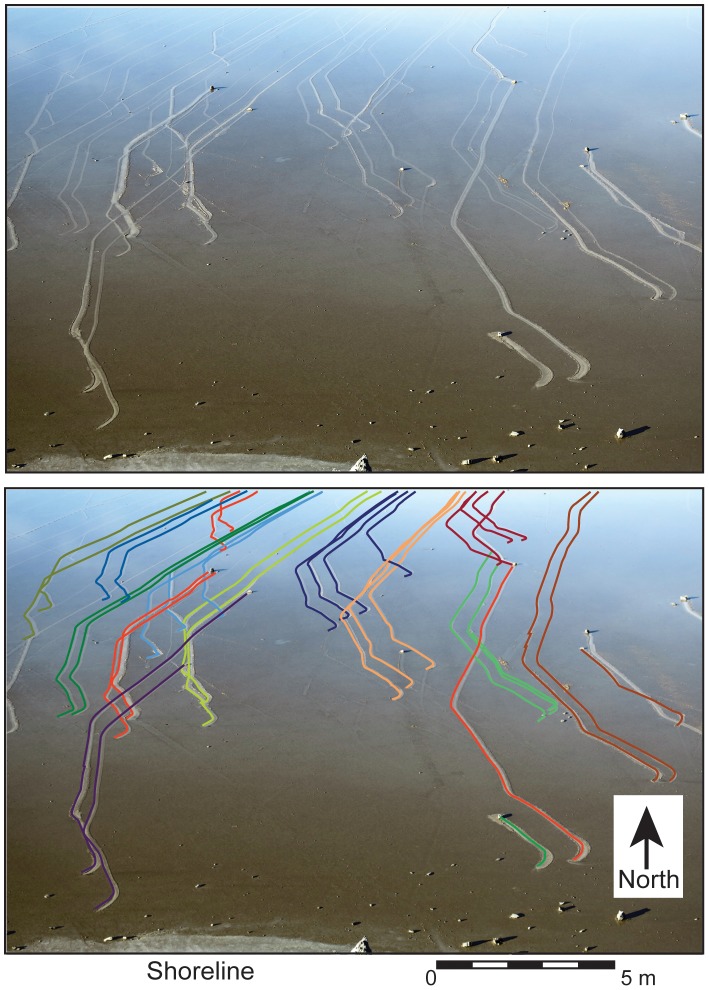
View from the ‘source hill’ on the south shore of Racetrack Playa. View is looking north on December 20, 2013 at 3:15 pm. Steady, light wind, 4–5 m/s has blown water to the northeast exposing newly formed rock trails. Lower image shows overlay of lines to emphasize the congruent shape of adjacent rock trails as well as the proximity of rock trails to rocks that did not move. Image has not been enhanced.

Racetrack Playa in Death Valley National Park, is well known for the phenomenon of tracks left by hundreds of rocks plowing across the nearly flat playa surface (Fig. 1). Rock movement by pebble to boulder-size pieces of dolomite and granite leaves tracks in the playa surface showing the direction of motion via groves cut in the playa mud. Remarkably, multiple rocks commonly show parallel tracks (Fig. 2), including apparently synchronous high angle turns and sometimes reversals in travel direction [1], [2], [3], [4]. The phenomenon of rock motion has excited considerable interest, and there is a scientific and popular literature extending back to the first report in 1948 [1], [2], [3], [4], [5], [6], [7], [8], [9], [10], [11], [12], [13]. Since then, theodolite mapping surveys, repeat photography and, most recently, the use of high resolution submeter GPS to map the rocks and their trackways have shown that the rocks move very episodically, often with no motion for several years to a decade or more [1], [2], [3], [4]. Various mechanisms for rock motion have been proposed, but owing to the harsh nature of the playa surroundings, and the difficulty of access, there has been no unambiguous determination of the mechanisms for rock motion.

All authors agree that rocks are most likely to move when the playa surface is wet, creating a slick surface, and that wind must be involved. The first scientific study of the Racetrack suggested that rock motion was driven by dust devils [5]. This idea was tested using the wash of an aircraft propeller over wetted surfaces of Racetrack Playa [13]; these experiments showed that winds more than 20 m/s could move natural rocks. Shelton [13] suggested that other factors, including the presence of algal films might help to lower the frictional forces resisting rock motion under strong winds. W. Sharp [14] carried out static and dynamic friction tests using rocks towed across wet and dry mud surfaces and calculated that wind velocities of 33–45 m/s would be needed for rock movement. Additional calculations for rocks of various sizes and sail heights showed that most rocks would move across a wetted playa surface where the coefficient of static friction is about 0.15 and wind velocities were >40 m/s [7]. Still other static friction tests suggest the need for even higher wind velocities (up to 80 m/s), particularly to move rocks with relatively low profiles [2]. All these experiments suggest that very high winds are needed for rock movement.

Other authors, led first by Stanley [4], argued that rocks are frozen into sheets of ice that reduce the friction with the underlying lake bed and increase wind drag [4], [8], [10], [11]. Most of these authors also note that multiple rocks can follow almost identical tracks, suggesting that they were moved while frozen onto a large layer of ice floating on liquid water. Reid et al. [2] made extensive observations of rock trails and showed parallel movements between rocks up to 830 m apart, implying very large sheets of ice. These authors also noted that parallel trails can involve rocks of different sizes that usually do not rotate or tumble during movement—both observations that suggest ice, rather than wind alone, is responsible for rock movement. It has also been noted that rocks encased in ice can actually partly float off the surface of the playa mud leaving shallower tracks than would be expected for a rock moving by wind alone across a muddy surface [8], [11].

In a test of the ice sheet hypothesis, R. Sharp and Carey [3] performed a now famous “corral” experiment, in which they drove a series of stakes into the playa surface around several rocks. The goal was to test whether the rocks would move independently as might be the case for wind-driven movement in the absence of ice. One rock moved out of the corral during the next winter while another rock remained inside the circle of stakes—a pattern Sharp and Carey [3] interpreted as evidence that ice is not the driving mechanism for rock motion. Finally, Messina and Stoffer [1] mapped the locations of the rocks and traced the visible trails using submeter differential GPS. Although there are broad similarities in the tracks of many rocks, deviations in trails suggest that the rocks were likely moving independently of one another rather than being propelled by a single ice sheet [1].

## Methods

**Figure 3:**
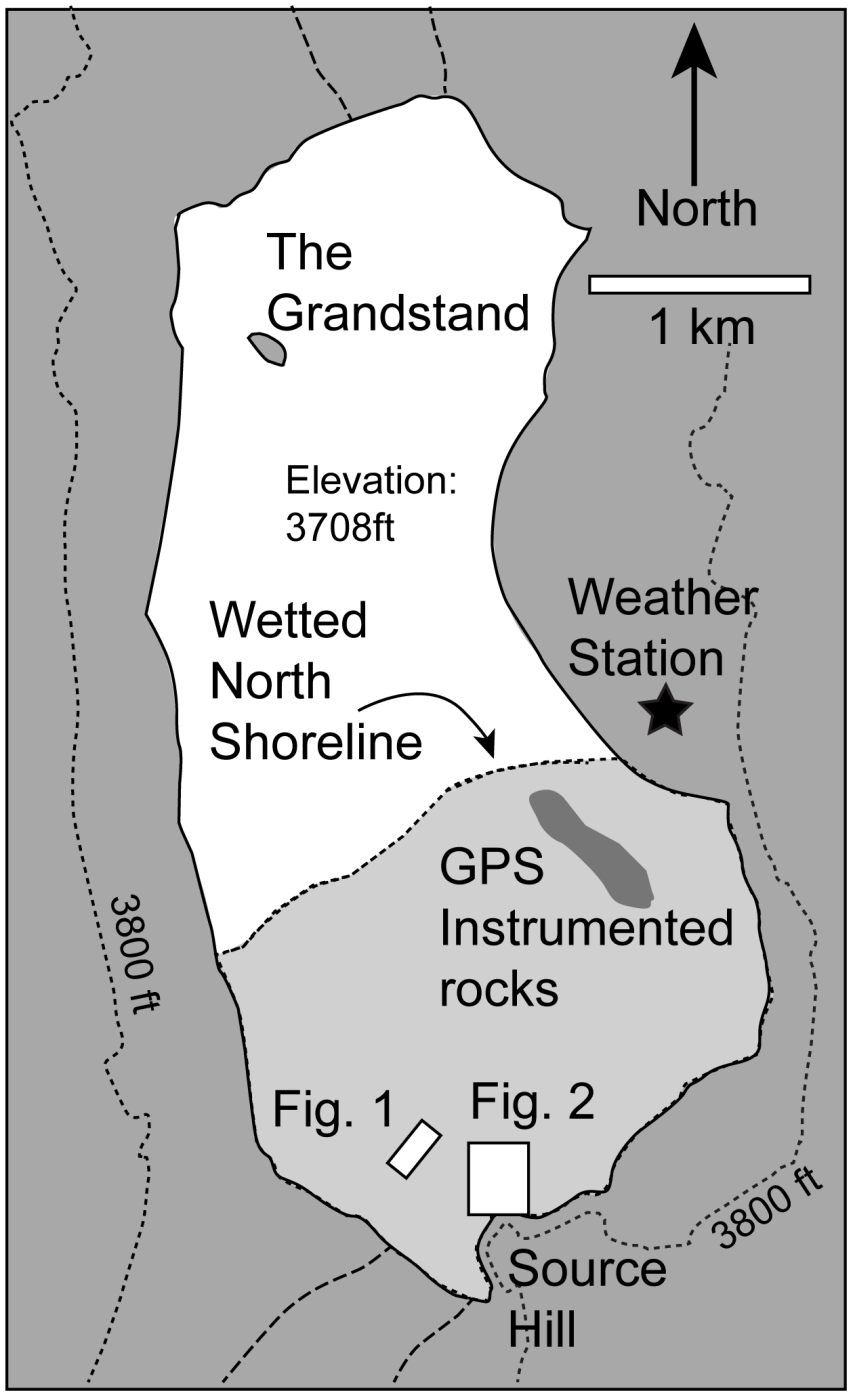
Map of Racetrack Playa. Map shows the locations of the weather station, GPS-carrying rocks, and approximate locations of the northern shoreline of the pool and Figures 1 and 2.

To describe the meteorological conditions on the playa and the velocities and timing of rock motion we installed a weather station adjacent to Racetrack Playa, several time lapse camera systems overlooking the southeast corner of the playa [9], and 15 GPS-instrumented rocks on the playa surface (Fig. 3). We visited the playa 5–8 times a year to exchange battery packs and download weather data. The time lapse camera was set up to record conditions hourly between November and March, each year [9]. The weather station and GPS-instrumented rocks were installed under Wilderness research permits DEVA-65173, DEVA-2012-SCI-0021, DEVA-2011-SCI-0047 and DEVA-2010-SCI-0023 from Death Valley National Park. Time lapse cameras were installed under NPS studies DEVA-00169 and DEVA-00341.

The weather station was obtained from the Sweeney Granite Mountains Desert Research Center (part of the University of California Natural Reserve System) and continuously recorded wind speed at 1 second intervals, along with temperature, insolation, rainfall, and GPS location. We anchored the weather station to the alluvial fan surface with sand bolts at N36.6823, W117.5515, northeast of the largest concentration of rocks on Racetrack Playa (Fig. 3). This location is along the trajectory of the longest rock trails mapped by Messina and Stoffer [1]. The weather station was equipped with Campbell Scientific CR-800 Data Logger, CS LI200X-LC Pyranometer, CS HMP35C-LC Temp and RH probe, CS GPS16X-HVS-PW, and a R.M. Young 05103-5 Wind Monitor. Rainfall collection was made with a Campbell Scientific TE525-LC Tipping Bucket Rain Gauge; note that this device was not equipped to measure snowfall or snow-water equivalents. Wind speeds were measured at 1 second intervals explicitly to determine the peak velocity of gusts. Wind strengths at the surface of the playa (where the rocks are located) may not be well represented by our anemometer that was located 3 meters off the alluvial fan surface. Data are reported in Table S1.

**Figure 4:**
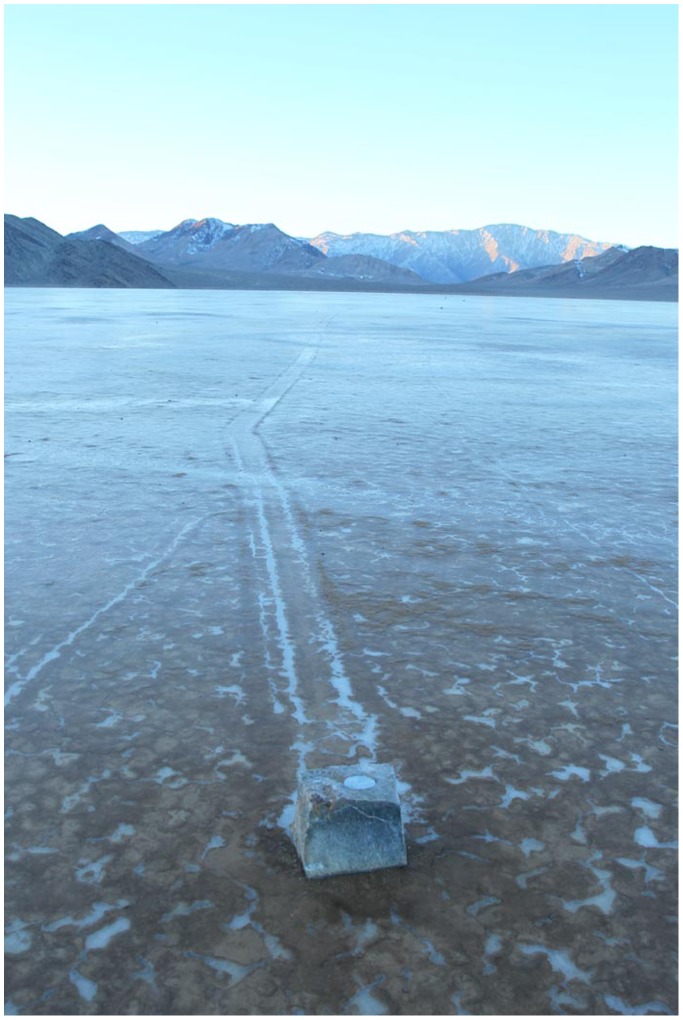
GPS-instrumented rock with its rock trail. The GPS unit with its battery pack is inserted into a cavity bored into the top of the rock. The GPS continuously logs its position after a switch is triggered by the stone moving away from a magnet set in the playa. The surface of the playa is frozen in this image, but the ice had melted or was floating when the trail formed. Image by Mike Hartmann.

Custom-built GPS loggers engineered by Interwoof were placed in limestone blocks of varying sizes and located on Racetrack Playa to the northeast of the largest concentration of natural stones (Fig. 4). Instrument packages recorded their GPS location and logger temperature at 60 minute intervals, and were designed to record continuously (at one second intervals) once they were disengaged from a magnetic trigger buried in the playa surface under each rock. Limestone blocks were obtained from the Panamint Springs Member of the Permian-aged Darwin Canyon Formation in Darwin Canyon, California (N36.28936, W117.53727), and were modified using a concrete boring tool to create a cavity for the GPS logger. Table 1 summarizes rock data for those GPS-instrumented rocks that moved during the deployment. Some of these rocks had flooded instrument packages and consequently we have data for only their starting and ending positions. Three rocks fully recorded their initial movement positions and velocities; these data are reported in Table S2. Two of these rocks moved again sometime after their initial movements (and after the GPS batteries had been depleted) and so developed total trail lengths longer than those shown in Table S2.

**Table 1:** Characteristics of GPS-instrumented rocks.

RockNo.	Mass (kg)	Starting latitude	Starting Longitude	Ending Latitude	Ending Longitude	Total trail length(m)
A2	∼9	36.67268	−117.55301	36.67415	−117.55231	174.7
A3	16.6	36.67341	−117.55299	36.67476	−117.55218	157.5
A5	6.7	36.67425	−117.55383	36.67595	−117.55248	224.0
A6	8.2	36.67415	−117.55452	36.67547	−117.55386	162.4
A9	11.7	36.67525	−117.55476	36.67538	−117.5547	15.4
A10	15.6	36.6749	−117.55598	36.67538	−117.55572	58.1
A11	15.4	36.67583	−117.55546	36.67614	−117.55528	39.1
A12	∼12	36.6762	−117.55453	36.67656	−117.55429	45.3

Rock mass (kg), starting position, ending position, and total length of movement (m) for eight of 15 GPS-instrumented rocks. The other seven rocks in the deployment did not move, or were not recovered by the time of manuscript submission. Rocks A3, A6 and A11 recorded their position and velocity during their initial movements as reported in Table S2. Trail end positions for all rocks other than A11 were recorded with a handheld consumer-grade GPS unit with ±5 m uncertainty. Rock A11 positions are recorded from its internal GPS unit.

## Results and Discussion

**Figure 5:**
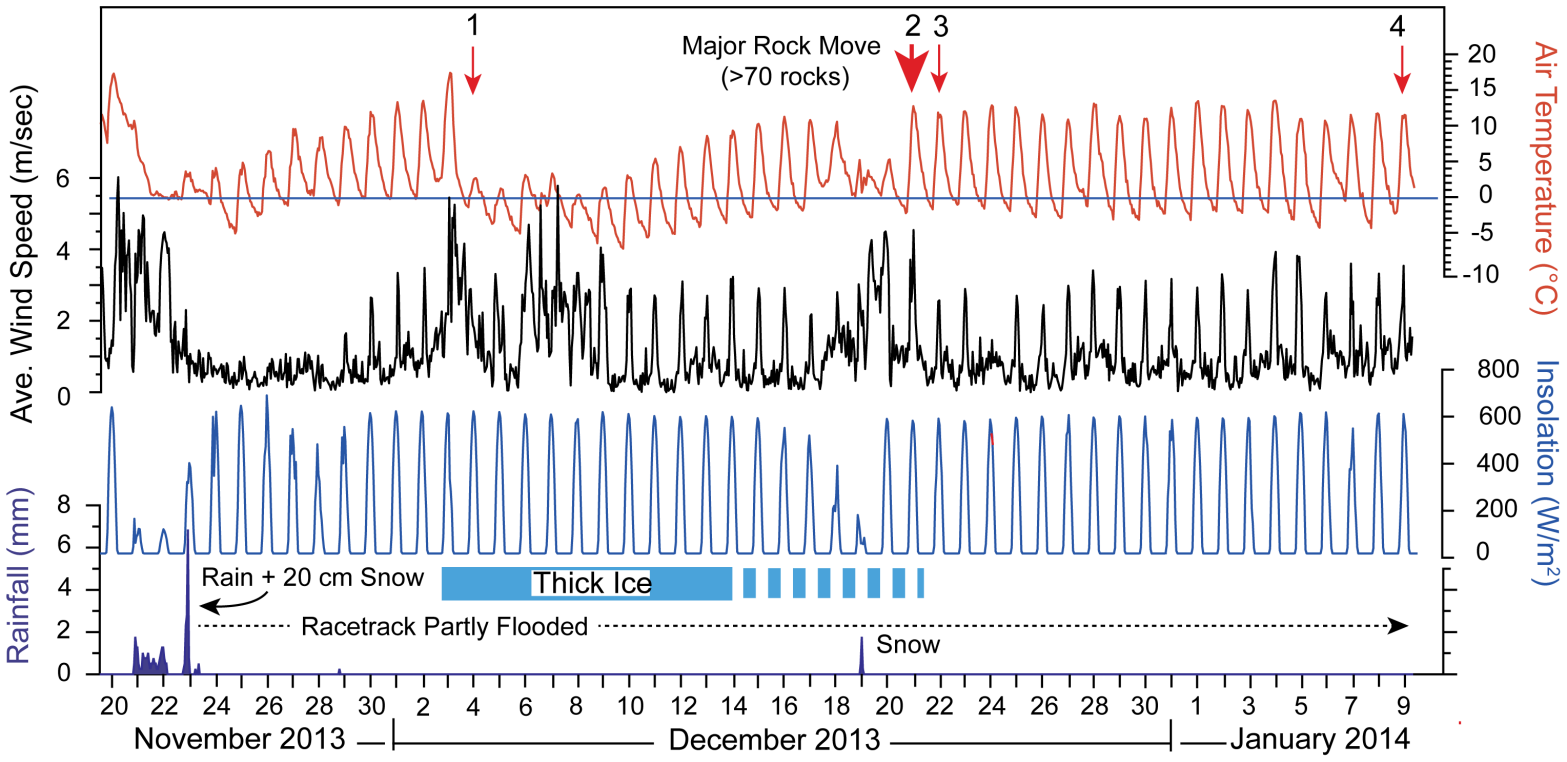
Weather record from Racetrack Playa, Death Valley National Park. Temperature (red line), 1 hour average wind velocity (black line), insolation (blue line), and rainfall (filled blue line) for November 20, 2013 to January 9, 2014. Wind gusts shown in Fig. 9. Red arrows indicate known rock movement events; there have been additional movement events not recorded by direct observations. Movement events 1 and 2 are recorded by instrumented rocks, and direct observations were made for events 2–4. Time lapse camera observation shows ∼20 cm of snow accumulation on November 23. Total precipitation is likely under-recorded because our weather station does not record precipitation due to snow. Data reported in Table S1.

We recorded rock movement associated with a shallow pond (∼10 cm maximum depth) that existed on Racetrack Playa between late November 2013 to early February 2014 (Fig. 5). Our weather station data and time lapse camera images revealed that the only significant rainfall was on November 21–24 when a combined total of 3.61 cm of rain, and ∼20 cm of snow fell during a regional winter storm (Fig. 5). Assuming a conservative snow/water equivalent for ∼0°C of 20 cm snow/2.03 cm liquid water based upon NOAA tables (reported at: www.erh.noaa.gov/box/tables/snowfall-meltwater.html), the total precipitation was ∼5.64 cm. The resulting pond repeatedly froze as nighttime temperatures dipped below freezing for most days to the end of our observations on January 9, 2014 when the pond still covered ∼1/4 of the playa surface. The pond eventually evaporated completely by the second week in February, 2014.

Observed rock movement occurred on sunny, clear days, following nights of sub-freezing temperatures. Steady light winds and morning sun caused floating ice to break-up near mid day, accompanied by widespread popping sounds from fragmenting ice panels. Ice initially broke into floating panels tens of meters in size that became increasingly fragmented and separated by open rippled water as melting continued. Floating ice sheets driven by wind stress and flowing water, pushed rocks resting on the playa surface, in some cases moving >60 rocks in a single event (Fig. 2).

Rocks move slowly and somewhat episodically during move events. For instance, on January 9, 2014, a rock was observed to move at ∼1–2 m/minute for about 18 seconds at 12:50 pm (Fig. 1). This rock was pushed by an ice panel estimated to be about 5–8 m long upstream of the stone.

**Figure 6:**
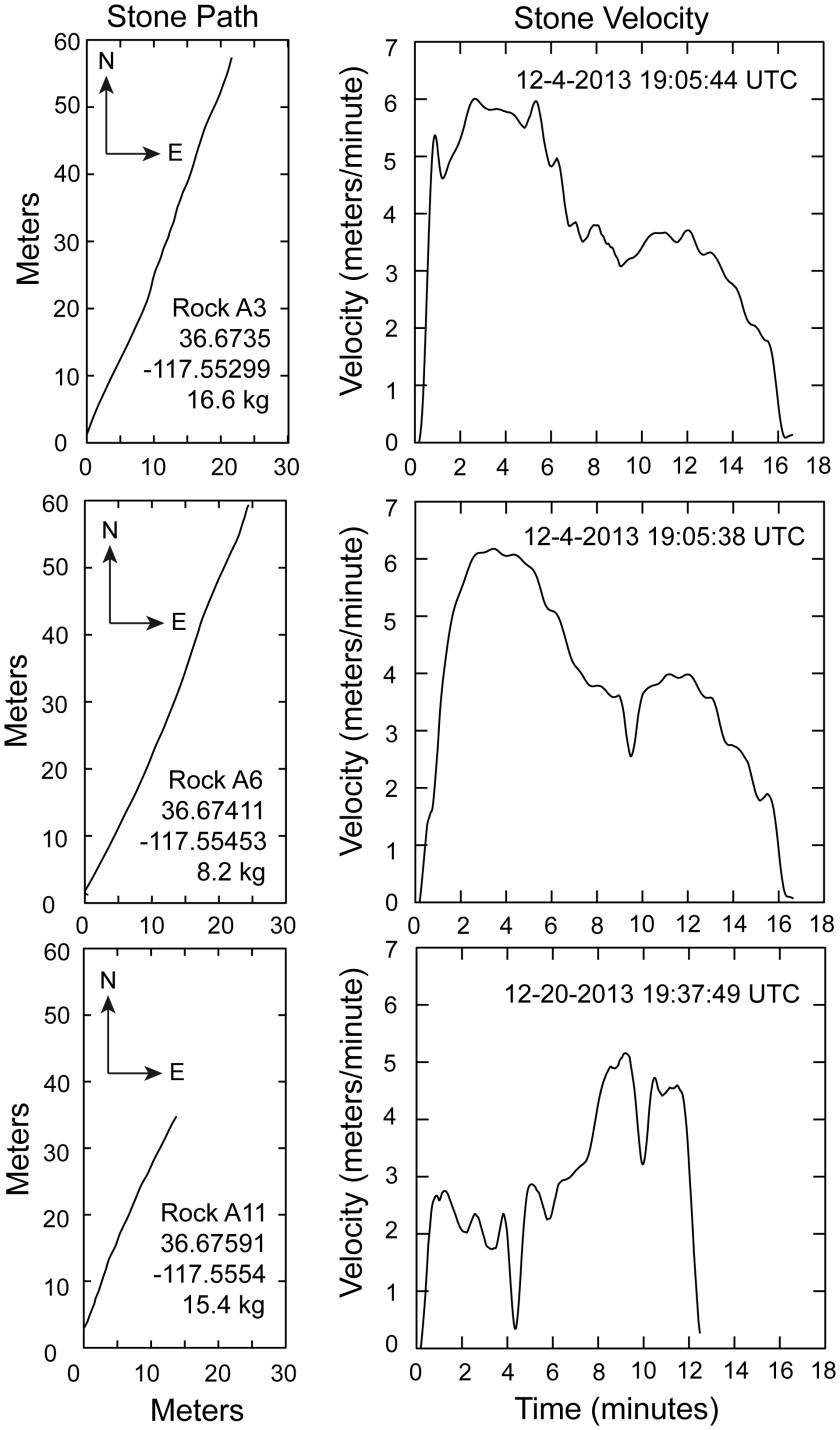
GPS-instrumented rock trajectories and velocity. Top and middle panels are data collected by rocks (A3 and A6) on December 4, 2013; bottom panels are for a rock movement on December 20, 2013 (Rock A11). Times are UTC. Note the broadly similar trajectories and velocity histories for rocks on December 4. Velocity errors are shown in Fig. 7. Data reported in Table S2. Rocks A3 and A6 moved at least once after their GPS instrument batteries were depleted and had total trail lengths of 157.5 m and 162.4 m, respectively (Table 1).

**Figure 7:**
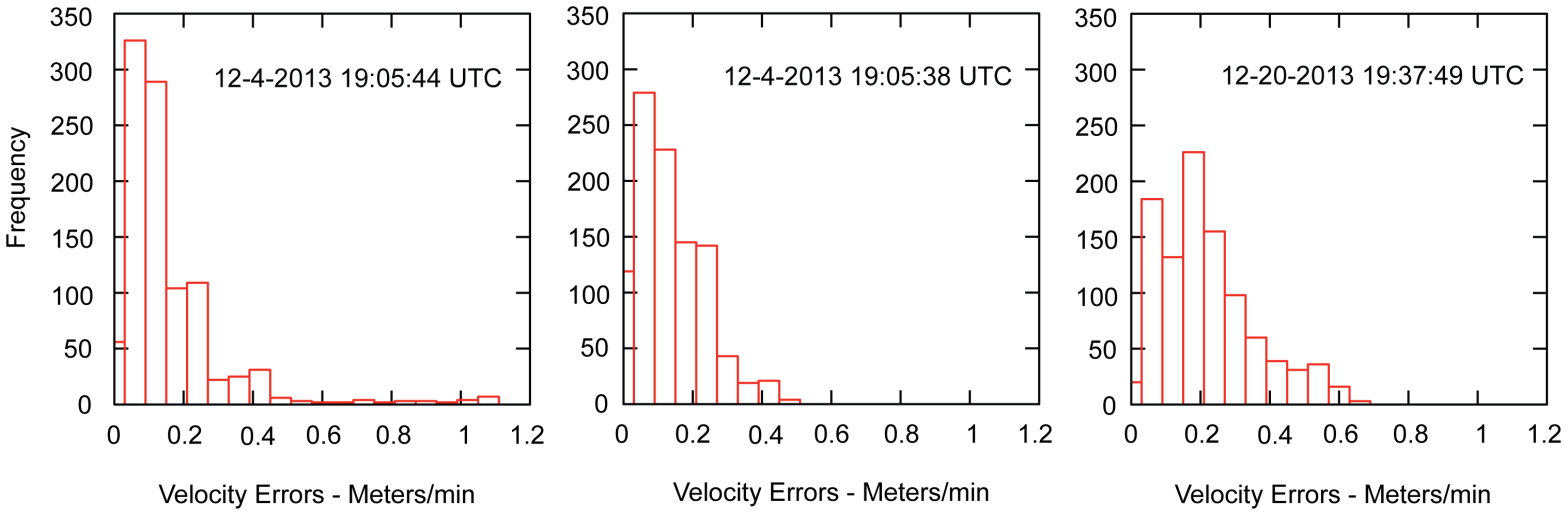
Error analysis of GPS-instrumented rock movement events. Note that the uncertainty in rock velocity is generally less than 0.2–0.3 m/minute in comparison with the recorded 2–6 m/minute velocities (Fig. 6).

Our instrumented rocks recorded movement events on December 4 and December 20, 2013 (Fig. 6). Two rocks recorded movements on December 4; one trail was 65.6 m long (A3; stone mass 16.6 kg) and the other of 64.1 m (A6; stone mass 8.2 kg). Both movements lasted 16 minutes starting at 11:05 am local time. These rocks were originally located ∼153 meters apart, and began motion within 6 seconds of each other. Both rocks initially reached velocities of 5–6 m/minute that fell to 3–4 m/minute by 6 minutes into the move event. The December 20 event is recorded by one rock (A11; stone mass 15.4 kg) with a 39.1 m movement over 12.3 minutes starting at 11:37 am. The rock initially achieved a velocity of 2–3 m/minute, then nearly stopped 4 minutes into the move, resumed a minute later, and traveled 5 m/minute to the end of the move event. Error analysis shows that for each rock, the velocity uncertainty is generally <0.3 m/minute (Fig. 7). Rock velocities are consistent with time-lapse images and observations on December 21 and January 9 (Fig. 1). In situ rock movements were detected by observing the position of moving rocks relative to stationary stones. However, the low velocities involved make it difficult to detect movement events by casual observation. Rock trails are formed under the ice, and become visible only when the muddy water is blown away by light winds.

**Figure 8:**
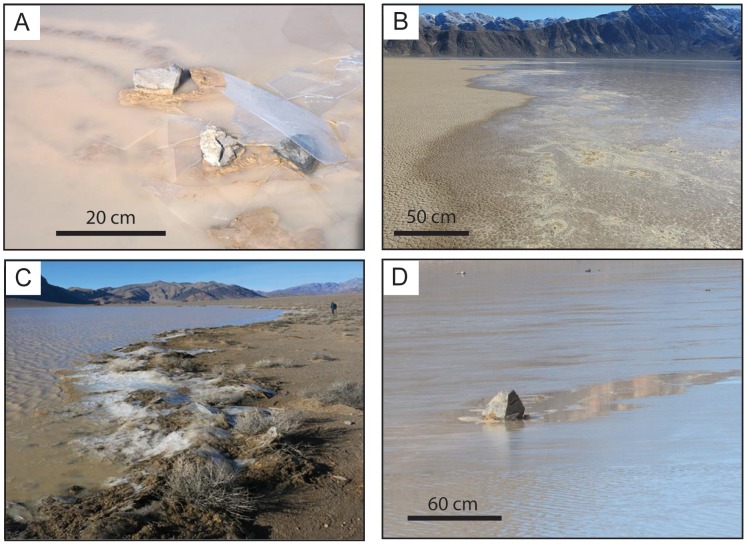
Racetrack Playa phenomena. Parts show: (a) thin windowpane ice over-riding a recently moved rock (January 9, 2014), (b) water creeping onto the low gradient northern shore of the pond during the December 20, 2013 rock movement (∼11:15 am), (c) ice windrows on the eastern shore of Racetrack Playa near the weather station (∼1 pm, December 20, 2013); ice panels are ∼2–3 mm thick and mixed with mud and rocks scoured from the pond bottom, (d) rock carving a wake through ice that is moving left to right; open rippled water in the foreground (January 9, 2014). Images have been cropped but not otherwise edited.

Floating ice often fragments upon encountering rocks, producing wakes of open water filled with ice chips downstream of both stationary and moving rocks (Fig. 8d). Ice panels also stack up on the upstream side of large rocks, increasing the effective surface area of rocks exposed to upstream ice as well as water flowing under the ice (Fig. 8a). The splintering of ice sheets can instantaneously decouple the movement of rocks, and may explain the movement of one rock and not another in Sharp and Carey's ‘corral’ experiment in which one rock moved out of a circle of stakes driven into the playa surface while one rock remained behind [3]. Indeed, there was a stake just upstream of the unmoving stone in the Sharp and Carey experiment that may have shattered a moving ice sheet before it encountered the stationary rock. Floating ice may sometimes be ineffective at moving rocks since stones with low profile may be over-ridden by floating ice; rocks at the edges of ice panels may fail to sufficiently engage with floating ice to be moved, and rocks may be too massive for the available force (Fig. 8d).

Ice movement produces rock trails that can be startlingly similar as seen in the December 20 event (Fig 2). Rock trails 50–60 m apart show broadly similar turns and segment lengths between turns although they may differ in the details. However, some rocks also moved past stationary rocks and the total travel distance varied by tens of meters for initially adjacent rocks. These contrasts in rock behavior are consistent with observations that fractures in the ice can decouple the movement histories of rocks only tens of centimeters apart, while congruent movements spaced many 10 s of meters apart can sometimes occur. The floating ice sheets at breakup are initially very large, so congruent rock trails may reflect motion early in the move event before large ice panels become fragmented, as proposed by Reid et al. [2]. However, partly correlated movement can also occur in rocks pushed by adjacent ice sheets since forces can still be transmitted under compression across fractures, explaining the partly parallel rock trials observed by Messina and Stoffer [1]. Finally, the water depth is a factor controlling which rocks move and which do not. Ice was observed to float or slide over low profile rocks, which may remain stationary while other adjacent, higher-profile, rocks move. Therefore, low profile rocks may have shorter trails (or not move at all) while higher profile rocks remain engaged with floating ice as they move into deeper water.

A surprise is the thinness of ice involved in rock movement. Ice sheets 3–6 mm thick are insufficient to float rocks off the playa surface, as proposed in some models [8], [11], and, in any case, we observed that ice melts first around rocks. However, moving sheets of ice tens of meters in extent but only a few millimeters thick are clearly effective at moving rocks in their path. Forces on stones increase when multiple sheets of ice pile on the upstream side of a rock and increase the effective surface area of the rock exposed to stresses of wind and flowing water (Fig. 8a). These ice piles are capable of scouring large amounts of mud and rocks from the lake bed onto the shoreline, as is well known from temperate lakes and rivers [15], [16]. Indeed, ice pile-ups (such as those documented from northern lakes [17]) have created 30–50 cm high sediment mounds on much of the southeast and southwest shorelines where the playa elevation is lowest and ponds are most persistent (Fig. 8c).

**Figure 9:**
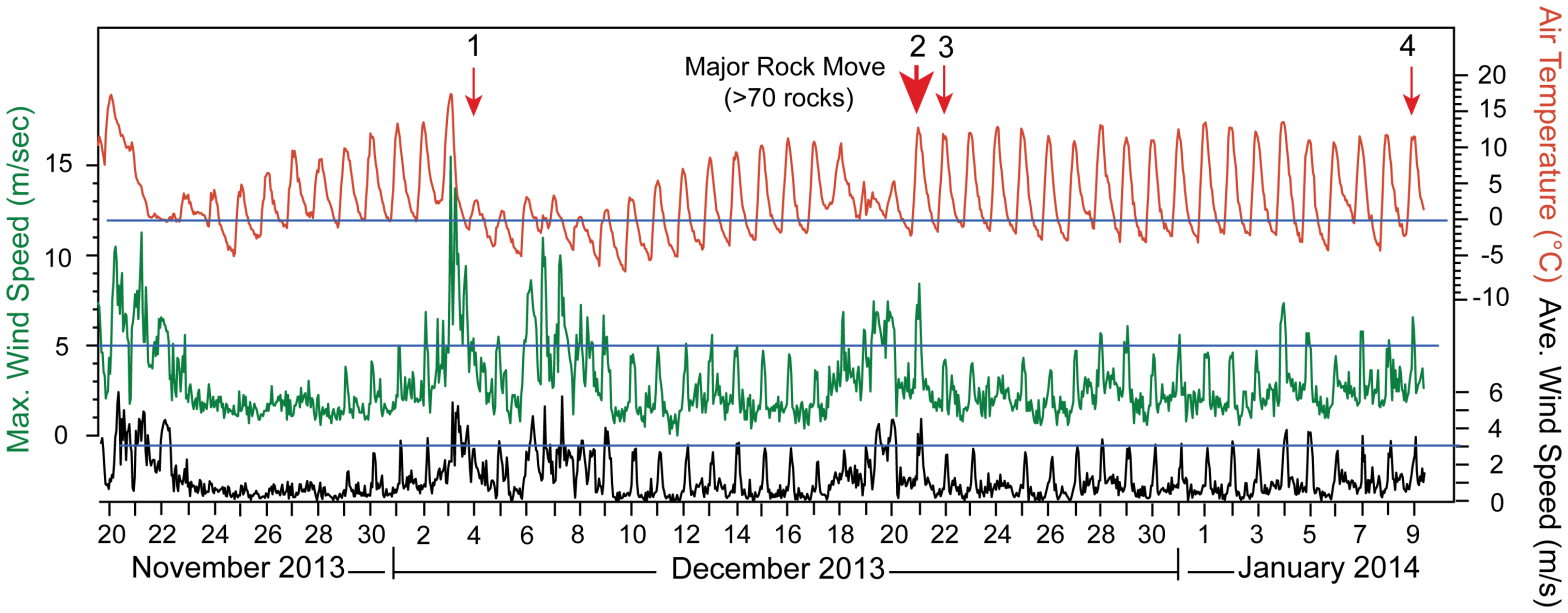
Gusts and average wind velocities. Comparison of average hourly wind velocity (black, as shown in Fig. 4) and hourly maximum wind velocity (green) against the daily temperature record (red line) and rock movement history (red arrows). Data reported in Table S1.

Rock movement is correlated with wind-driven transfer of water from the southern zone of the playa to the northern margin under sustained light winds of 3.0–4.5 m/s (Fig 5). These wind events included some stronger gusts during known rock movement events (up to 8.4 m/s, Fig. 9) but it seems likely that sustained winds are needed to keep both the ice and liquid water of the pond in motion before ice melts completely. On December 20, 2013, we observed water flooding the northern shoreline of the pond at 60–100 cm/minute in the mid morning, gradually transferring water from the southern region of the pond (where most of the rocks are located) to the northeastern part of the pond. Winds blew water in a seiche-like event onto the low gradient northern shoreline of the playa pond inundating it to a depth of 1–2 cm (Fig. 8b). The shallow depth of water on the northern shore, and albedo of the underlying mud of the pond, likely insured that ice melted here before widespread ice breakup occurred, creating a large expanse of liquid water for wind to act upon and an area of open water for ice to move to. In the December 20, 2013 event, the pond adjacent to the southern shore decreased from ∼7 cm depth of muddy, nearly opaque water in the morning hours, to <1 cm depth by 3:10 pm, revealing >60 fresh rock trails (Fig. 2) as the water was driven away northward by the light southerly winds.

Time-lapse images acquired since 2007, typically from late November to early March, show that the conditions we have observed are quite rare. Snowfall on January 2–3, 2011 and February 27, 2011 blanketed the playa for a couple of days but did not lead to flooding. Hills near the playa received a couple of dustings of snow in early 2012, but the south end of the playa did not experience more than a brief sheen of moisture. Winter 2012–2013 was likewise essentially dry. Although a ∼30 day period of flooding occurred in the late winter of 2010 [12], temperatures during this period were rarely below freezing and little ice was observed. The only period with observed conditions comparable to those during which we have presently observed rock motion is February, 10–15, 2009 [12]. A single, small trail was suspected to have formed during that season [11].

A persistent pond can support multiple movement events; our GPS instrumented rocks have recorded at least two separate move events with total trail lengths for a single stone of up to 224 m (Table 1). Our weather station adjacent to the playa recorded multiple nighttime freezing events and subsequent episodes of sustained daytime winds of 3.0–4.5 m/s, suggesting that there may have been more rock movement events than we have recorded during the ∼3 month lifetime of the 2013–2014 pond (Figs. 3, 8). Indeed, the persistence of the pond explains why we and others [2], [3], [4] have observed multiple trail segments for a single rock that are separated by ‘sitz’ marks, where the rock was apparently immobile for a period of time. The fresh appearance of many of these trail segments formed by one rock likely reflects the short period (days or weeks) between rock movements rather than movement events separated by many years. Likewise, changes in wind and water flow vectors between different days can explain the often high angle turns between different trail segments created by a single stone.

A surprising finding is the power of even thin sheets of ice to move large stones, without buoyant uplift [15], [16], [17]. We further note that most stones were ‘bulldozed’ with a sliding motion, rather than rolling [18] (see also Reid et al. [2]), perhaps because movement occurs with low friction on a completely saturated mud surface. Rock movement on Racetrack Playa is similar to the movement of rocks in deeper lakes and marine basins where ice break-up is a regular spring phenomenon. For example, the ice-driven movement of rocks, including large boulders, is known to produce rock trails on the shallow bottom of the Great Slave Lake in northern Canada [19] and the shores of the Baltic Sea [20]. Ice is also likely to explain rock trails over usually dry lake surfaces in Spain [21] and South Africa [22] where relatively high elevation and cold winters contribute to the formation of floating ice.

## Conclusions

A necessary condition for the rock motion we observed is the existence of a playa pool deep enough to submerge the southern section of the playa, yet shallow enough to leave many rocks partly exposed at the pond surface. Other repeating features of rock movement events that we observed include the presence of floating ice, temperatures and sunlight sufficient to create melt pools in the ice, and light breezes that are steady enough to drive floating ice. Although the ice breaks up around rocks, even thin moving ice sheets can generate sufficient force to drive rocks across the pool. All observed rock movement events occurred near mid-day when sufficient ice melting had occurred to allow ice break-up. Creation of rock trails is difficult to observe because trails form below the ice-covered pool surface where they are often not evident until the ice has melted, and liquid water has been removed. In addition, rock movement is slow and relatively brief—our GPS instrumented stones traveled at speeds of 2–5 m/minute for up to 16 minutes—so casual observation is likely to miss rocks in motion. Weather station data show that the freezing temperatures necessary for ice formation, and winds in excess of 3–5 m/s are common phenomena at Racetrack Playa during the coldest few weeks of winter. Therefore, the extremely episodic occurrence of rock motion (years to decades) is likely due to the infrequency of rain or snow events sufficient to form winter ponds.

## Supporting Information

Table S1 **Weather data collected from Racetrack Playa, Death Valley National Park.** Data period: Nov-20-2013 to Jan-9-2014. Records of hour-total rainfall (column 2), as well as hourly average insolation (column 3), air temperature (column 4), and wind velocity (column 5) with the time stamp given in column 1. The record of maximum wind gust strength (in column 7) is calculated to the nearest minute with a time stamp given in column 6. Station located at N36.6823, W117.5515. Instrument package specifications reported in the text and table header.
(CSV)

Table S2 **Movement data for GPS-instrumented rocks on Racetrack Playa, Death Valley National Park.** Data obtained for three rocks (A3, A6, and A11) that recorded position and velocity. For each rock, movement data are date (column 1), time stamp (to nearest second UTC, column 2), latitude (degrees, column 3), longitude (degrees, column 4), and velocity (m/minute, column 5). Rocks A3 and A6 had total trail lengths longer than recorded by their GPS instrument packages (Table 1), showing that they moved at least one more time after their GPS batteries had been depleted. GPS instrument packages are custom designed units by Interwoof.
(CSV)
